# Factors Predicting Uptake of Sexually Transmitted Infections Testing among Men Who Have Sex with Men Who Are “Pre-Exposure Prophylaxis Tourists”—An Observational Prospective Cohort Study

**DOI:** 10.3390/ijerph18073582

**Published:** 2021-03-30

**Authors:** Zixin Wang, Yuan Fang, Natthakhet Yaemim, Kai J. Jonas, Andrew Chidgey, Mary Ip, Tommy Cheng, Joseph T. F. Lau

**Affiliations:** 1 JC School of Public Health and Primary Care, Faculty of Medicine, The Chinese University of Hong Kong, Hong Kong, China; mitk@cuhk.edu.hk (M.I.); kwanwacheng@gmail.com (T.C.); 2 Department of Early Childhood Education, The Education University of Hong Kong, Hong Kong, China; lunajoef@gmail.com; 3 Pulse Clinic, Bangkok, Thailand; ntk1987@gmail.com; 4 Department of Work and Social Psychology, Maastricht University, P.O. Box 616, 6200 MD Maastricht, The Netherlands; kai.jonas@maastrichtuniversity.nl; 5 AIDS Concern, Hong Kong, China; Andrew.chidgey@aidsconcern.org.hk

**Keywords:** PrEP tourists, men who have sex with men, STI testing, observational prospective cohort study

## Abstract

The term “Pre-exposure prophylaxis (PrEP) tourists” refers to individuals who obtain PrEP in other countries and use it in their home countries. A prospective observational cohort study was conducted among a group of men who have sex with men (MSM) who obtained PrEP in private clinics in Thailand and used it in Hong Kong. Participants completed two web-based self-administered surveys when obtaining PrEP in Thailand and three months afterwards. Out of 110 participants at baseline, 67 completed the follow-up. The prevalence of sexually transmitted infection (STI) testing was 47.8% during the follow-up period. Eleven participants received an STI diagnosis, and seven of them were incident infections in the past three months. Participants who perceived a higher chance for STI infection (adjusted odds ratios (AOR): 1.90, 95% CI: 1.00, 3.75) and reported higher intention to take up STI testing at baseline (AOR: 1.62, 95% CI: 1.05, 2.50) were more likely to receive STI testing during the follow-up period. Baseline perceptions that service providers would think they were having risky behaviors because of PrEP use was negatively associated with the dependent variable (AOR: 0.51, 95% CI: 0.31, 0.86). Service planning and health promotion related to STI testing is needed for MSM “PrEP tourists”.

## 1. Introduction

Pre-exposure prophylaxis (PrEP) is an evidence-based risk reduction measure for men who have sex with men (MSM), which refers to the initiation of Tenofovir Disoproxil Fumarate/Emtricitabine before and during periods of HIV exposure among HIV-negative individuals in order to prevent HIV acquisition [1]. Daily PrEP with good adherence can reduce the risk of HIV infection by over 90% among various at-risk groups [1,2,3]. Recent evidence showed that on-demand PrEP, which refers to taking two pills between 2 and 24 h before sex and two pills 24 and 48 h after the first dose, could reduce the risk of HIV acquisition by 86% among MSM [4]. The World Health Organization (WHO) strongly recommends daily PrEP to all populations at substantial risk of HIV infection [5]. The WHO also states that on-demand PrEP can be offered as an alternative to daily PrEP to MSM as part of comprehensive HIV prevention and sexual health services [6].

Currently, only a few countries (e.g., Norway, Scotland, France, and Kenya) have established national policies for free PrEP; a few other countries (e.g., Australia, New Zealand, Belgium, Canada, Thailand, and South Africa) are subsidizing people at high risk of HIV [7,8,9]. In Hong Kong, PrEP is neither free nor subsidized. The coverage of the PrEP demonstration project in Hong Kong was limited, and the cost to obtain PrEP from private clinics is quite high (approximately US $1000 per month) [10]. MSM in Hong Kong showed demands for PrEP [10,11,12]. Due to the high cost, the majority of local MSM PrEP users obtained the medication from overseas clinics or through online purchase [10,13]. There is an emerging phenomenon of “PrEP tourists”, which refers to individuals who obtain PrEP in other countries and use it in Hong Kong. This paper seeks to document sexually transmitted infection (STI) testing behaviors among such PrEP tourists, in order to tailor future interventions to serve this population.

Although the benefits of PrEP in preventing HIV are widely recognized, there are concerns that PrEP use will encourage sexual risk behaviors and hence result in the spread of other STI. Early studies examining the impact of PrEP on STI risk yielded mixed results. Randomized controlled trials evaluating PrEP efficacy observed a decrease in sexual risk behaviors among participants at follow-up evaluations [1,3]. An open label extension trial of iPrEX and a demonstration study in the United States found no increase in STI infection among PrEP users [14]. As PrEP use became more common, studies suggested that many MSM reduced condom use after initiating PrEP. The prevalence of STI among MSM PrEP users ranged from 14.2 to 50.6%, while the STI incidence in this group was as high as 34.2–119.8 per 100 person-year [15,16,17,18,19]. A recent meta-analysis suggested that PrEP use was associated with a 24% increase in the prevalence of STI and a 59% increase in the risk of rectal STI among users [15]. Although STI among MSM are seldom life-threatening, they are morbid and associated with increasing medical cost, and likely to increase risk of HIV transmission [20]. Therefore, the PrEP clinical practice guideline issued by the United States Centers for Disease Control and Prevention (CDC) highlights the importance of regular STI testing for PrEP users [21]. It states that STI testing should be conducted at least every three months for sexually active PrEP users with signs or symptoms of infection and for asymptomatic MSM at high risk of recurrent bacterial STI (e.g., those with history of STI at prior visits, comdomless anal intercourse (CAI) or multiple sex partnerships) [21]. In line with the CDC, the Hong Kong interim statement on PrEP also emphasizes that screening and treatment of STI should be done regularly among users [22].

Assuming PrEP users are 100% complying with regular STI screening, an increase in PrEP coverage would reduce STI incidence at population-level after taking potential decrease in condom use after PrEP initiation into account [23]. This is because more asymptomatic STI patients are identified and linked to treatment during regular screening [23]. However, prevalence of STI testing was relatively low among MSM PrEP users. One study conducted in the United States showed that only 43.1% MSM received STI testing according to the CDC guideline at PrEP initiation, the STI testing rate dropped to 28.2% and 41.5% six and twelve months after PrEP initiation [16]. Another study in the United States showed that 69.3% of HIV-negative men who reported PrEP use in the past year had taken up STI testing [24]. Factors associated with STI testing among MSM PrEP users included those related to socio-demographics (e.g., ethnicity), sexual behaviors prior to PrEP initiation and during PrEP use, and whether healthcare providers offered HIV and STI testing [16,24]. These variables were considered by this study. However, there are no studies investigating STI testing behaviors among MSM PrEP users in the context of Hong Kong.

There was a large scale ongoing PrEP project (PrEP30) in Thailand that lasted from December 2014 to January 2021, which had established clinics that provided PrEP to foreigners at US $30 per month [25]. Given geographic accessibility, its positioning as a tourist location in general, and the low cost for PrEP, Thailand becomes one of the most popular destinations for Hong Kong MSM “PrEP tourists” [11]. Since most “PrEP tourists” only visit overseas PrEP clinics once, irregularly for refill of PrEP; it is difficult for them to obtain support from such clinics when they are back to Hong Kong. In Hong Kong, free STI testing and treatment services are provided by public social hygiene clinics, while such services are also available at a relatively high cost (US $100–300 per episode) in private clinics [26]. Some local non-governmental organizations (NGO) provide free STI testing services (i.e., syphilis, chlamydia and gonorrhea) to MSM, and they can refer STI patients to receive treatment in either public or private hospitals [27]. However, there are no STI testing services tailored to the needs of “PrEP tourists”. This may cause potential issues such as not taking up the STI testing regularly and lead to harm among this group of PrEP users.

Understanding facilitators and barriers to take up STI testing among “PrEP tourists” is important to inform intervention development and service planning. Perceptions had significant influence on HIV and STI testing behaviors among MSM, and they are modifiable through interventions [28,29,30]. Theory-based interventions are more effective than non-theory-based ones [31]. In this study, we applied the Health Belief Model (HBM) as the theoretical framework [32]. It postulates that perceived susceptibility and perceived severity related to STI, together with perceived benefit, perceived barrier, cue to action and self-efficacy related to STI testing are determinants of the behavior. The HBM is commonly used in explaining and promoting HIV or STI testing behaviors among MSM [28,29,30].

To address the knowledge and service gaps, this study investigated predictors of STI testing uptake within a three-month follow-up period among a sample of Hong Kong MSM “PrEP tourists”. Potential associated factors included socio-demographics and perceptions related to STI testing based on the HBM measured at baseline, sexual risk behaviors measured at both baseline and Month three, and methods of PrEP use during the follow-up period.

## 2. Methods

### 2.1. Study Design

A prospective cohort study was conducted among a group of MSM who obtained PrEP in Thailand and used it in Hong Kong during March 2018 to April 2019. Participants completed a web-based self-administered survey when obtaining PrEP in two participating clinics in Bangkok, Thailand. A total of 90 tablets of PrEP are prescribed at a time to users by these clinics. Therefore, we invited participants to complete another web-based self-administered survey three months afterwards.

### 2.2. Participants and Data Collection

Inclusion criteria included: (1) men of age 18 years or above, (2) had had anal intercourse with at least one man in the last six months, (3) obtained PrEP in two participating private clinics (two locations of PULSE Clinic) in Bangkok, Thailand, and used PrEP in Hong Kong, (4) able to read and comprehend Chinese and/or English, (5) having a Hong Kong ID Card, and (6) willing to leave contact information (e.g., mobile phone number, email, or social media account) and complete the follow-up evaluation at Month 3. There are five clinics currently providing PrEP to MSM from Hong Kong. The costs of obtaining PrEP and services are similar across these clinics.

The medical staff at the participating clinics screened the eligibility of clients who came to obtain PrEP based on the aforementioned six inclusion criteria. They briefed prospective participants by using an information sheet (in either Chinese or English), and invited them to participate. Guarantees were made of anonymity, right to quit at any time, and that refusal would not affect their chance to use any services. Prospective participants were invited to complete a baseline web-based self-administered questionnaire on a tablet at the participating clinics in Bangkok. After signing an electronic consent form and answering three questions reconfirming their eligibility (i.e., “Are you aged 18 years or above?”, “Are you living in Hong Kong?”, and “Is it the first time for you to fill in this questionnaire?”), participants started the web-based survey which took about 20 min to complete. At the end of the web-based survey, they were asked to provide their contact information. An individual link to access the questionnaire was sent to participants via SMS/email/social media three months after completion of the baseline survey. For participants who failed to complete the questionnaire within one week, four weekly reminders would be sent to them in the same manner before they were considered as drop-outs. A Café e-coupon (HK $50 or US $6.40) was given to participants upon completion of each survey. Among the 175 participants who consented to join the study during the recruitment period (March 2018 to January 2019), 154 were confirmed to be eligible, and 110 completed the baseline questionnaire. The completion rate was 71.4%. A total of 67 participants completed the Month 3 follow-up questionnaire, the loss-to-follow-up rate was 39.1%. Ethics approval was obtained from the Survey and Behavioral Research Ethics Committee of the Chinese University of Hong Kong (ref#: MSS274R).

### 2.3. Measurements

#### 2.3.1. Design of the Questionnaires

A panel consisting of a public health researcher, an epidemiologist, two health psychologists, one private physician, and one NGO worker was formed to develop the questionnaire. The questionnaire was tested among 5 MSM PrEP tourists. Based on their feedback, the questionnaire was finalized by the panel.

#### 2.3.2. HIV, Hepatitis B Virus (HBV)/Hepatitis C Virus (HCV), Other STI and Renal Function Testing

At baseline, participants reported whether they have used any type of HIV testing, HBV and/or HCV testing, testing for any STI other than HIV, HBV and HCV (i.e., chlamydia, syphilis, genital warts, genital herpes, and other STI) and renal function testing in the past two weeks. Their utilization of any type of HIV testing, HBV and/or HCV testing, other STI testing and renal function testing during the 3-month follow-up period were measured at Month 3. Those self-reporting that they had taken up such testing were asked about their testing results.

#### 2.3.3. Baseline Background Characteristics

Information collected included socio-demographics (age, whether they were Hong Kong permanent residents, ethnicity, highest education level attained, relationship status, and monthly personal income), sexual orientation, duration of PrEP use and HBV vaccination uptake.

#### 2.3.4. Sexual Behaviors at Baseline and Month 3

The number of male regular sex partners (RP) and nonregular sex partners (NRP) with anal intercourse, CAI with men, and sexualized drug use (SDU) in the last three months were measured at baseline and Month 3. RP was defined as their lovers or stable boyfriends, while NRP was defined as men who were not their RP, including casual sex partners and male sex workers. SDU was defined as the use of any psychoactive substances (i.e., ketamine, methamphetamine, cocaine, cannabis, ecstasy, Dormicum/Halcion/Erimin 5/non-prescription hypnotic drugs, heroin, codeine from cough suppressants, amyl nitrates (poppers), γ-hydroxybutyrate (G water), and Foxy) before or during sexual intercourse [11,33].

#### 2.3.5. Methods of PrEP Use during the Follow-Up Period Measured at Month 3

At Month 3, participants were asked about their methods of PrEP use (daily PrEP, on-demand PrEP or episodic PrEP) during the follow-up period. Daily PrEP was defined as using PrEP once every day no matter whether you had anal intercourse or not, on-demand PrEP was defined as only using PrEP before and after anal intercourse, while episodic PrEP referred to using PrEP either daily or on-demand only in some period of time [34].

#### 2.3.6. Perceptions Related to STI Testing Measured at Baseline

Six items were constructed to measure perceptions related to STI testing based on the HBM. Perceived susceptibility to STI when using PrEP was measured as a single item “How high is your risk of contracting STI when using PrEP” (response categories: 1 = very low, 2 = low, 3 = moderate, 4 = high, and 5 = very high). Participants were also asked whether they agreed with the statements related to perceived benefit (i.e., “Taking up STI testing regularly when using PrEP could detect STI earlier, so as to have better treatment outcomes”), perceived barriers (“Service providers would think you are having risk behaviors because you are using PrEP” and “Taking up STI testing regularly when using PrEP is troublesome for you”), cue to action (“People who are important to you suggest you to take up STI testing regularly when using PrEP”), and perceived self-efficacy (“It is easy for you to take up STI testing regularly when you are using PrEP”) (response categories: 1 = strongly disagree, 2 = disagree, 3 = neutral, 4 = agree, and 5 = strongly agree). In addition, participants were also asked their chance of taking up any STI testing in the next 3 months (response categories: 1 = very low, 2 = low, 3 = moderate, 4 = high, and 5 = very high).

### 2.4. Statistical Analysis

Baseline characteristics of participants who completed Month 3 follow-up and those who were lost-to-follow-up were compared by using Chi-square tests (for categorical variables) or independent sample t-tests (for continuous variables). Subsequently, a number of analyses were performed with data from those who had completed both surveys. Using STI testing uptake during the follow-up period as the dependent variable, baseline background characteristics, HIV, HBV and/or HCV, other STI and renal function testing at baseline, HIV, HBV and/or HCV and renal function testing at Month 3, sexual behaviors at baseline and Month 3, and methods of PrEP use during the follow-up period as independent variables, crude odds ratios (OR) predicting the dependent variable were obtained by using logistic regression models. After adjustment for those variables with *p* < 0.05 in the univariate analysis, associations between independent variables of interest (perceptions related to STI testing) and the dependent variable were then assessed by adjusted odds ratios (AOR). Each AOR was obtained by fitting a single logistic regression model, which involved one of the independent variables of interest and significant background variables. SPSS version 21.0 was used for data analysis, with *p* values < 0.05 taken as statistically significant.

## 3. Results

### 3.1. Background Characteristics, Sexual Behaviors, and HIV, HBV and/or HCV, Other STI and Renal Function Testing, and Perceptions Related to STI Testing Measured at Baseline

The majority of the participants were younger than 40 years (82.7%), Hong Kong permanent residents (64.5%), Chinese (68.2%), currently single (54.5%), had attained tertiary education (91.8%), and self-identified as gay men (90.9%). Over one third of them had monthly income over HK$50,000 (about US $6400) (36.4%) and were first-time users of PrEP (39.1%). The prevalence of HBV vaccination was 80.9%. Among the participants, 100% had taken up HIV testing, 42.7% had taken up HBV and/or HCV testing, 62.7% had taken up other STI testing, and 75.5% had taken up renal function testing 2 weeks prior to the baseline survey. Regarding sexual behaviors, 34.6% and 70.9% had anal intercourse with more than one RP and NRP; 86.4% and 40.9% reported CAI with men and SDU in the last three months prior to the baseline survey.

Only 12.7% of the participants perceived a high or very high chance of contracting STIs. The majority of them (94.5%) agreed that taking up STI testing regularly was beneficial for STI early detection and treatment, while about 30% perceived some barriers to using STI testing when using PrEP. Over 75% were suggested by significant others to receive STI testing regularly, and 82.7% were confident to take up STI testing if they wanted. Over half (53.6%) of the participants intended taking up STI testing in the next 3 months. Mean and standard deviation (SD) of the aforementioned items are presented in Table 1.

As compared to participants who were followed up at Month 3 (n = 67), those who were loss-to-follow-up (n = 43) were more likely to be bisexual (*p* = 0.002) and reporting SDU at baseline (*p* = 0.03). (Table 1).

### 3.2. HIV, HBV and/or HCV, Other STI and Renal Function Testing during the Follow-Up Period

Among participants who completed the Month 3 survey (n = 67), 100% had taken up HIV testing, 6% had taken up HBV and/or HCV testing, and 47.8% had taken up other STI testing during the follow-up period. (Table 2).

None of them was tested to be HIV positive, while eleven of them received an STI diagnosis (chlamydia: n = 2; syphilis: n = 2; genital warts: n = 1; genital herpes: n = 1; gonorrhea: n = 5). Seven out of these eleven cases were incident infections in the past 3 months (chlamydia: n = 2; genital warts: n = 1; genital herpes: n = 1; gonorrhea: n = 3). About half (52.2%) of the participants reported renal function testing uptake during the follow-up period. Two participants received results indicating impaired renal function (data not presented in the tables).

### 3.3. Methods of PrEP Use and Sexual Behaviors during the Follow-Up Period

Among participants who completed the Month 3 survey (n = 67), 70.1% used daily PrEP, 26.9% used on-demand PrEP and 3% used episodic PrEP during the follow-up period. Regarding sexual behaviors, 31.4% and 67.1% had anal intercourse with more than one RP and NRP; 86.4% and 40.9% reported CAI with men and SDU in the last three months. (Table 2).

### 3.4. Factors Associated with STI Testing Uptake during the Follow-Up Period

Baseline STI testing significantly predicted STI testing uptake during the follow-up period (OR: 2.83, 95% CI: 1.00, 8.01, *p* = 0.049). After adjustment for this significant variable, participants who perceived higher susceptibility to STI (AOR: 1.90, 95% CI: 1.00, 3.75, *p* = 0.047), and reported higher intention to take up STI testing in the next 3 months (1.62, 95% CI: 1.05, 2.50, *p* = 0.03) were more likely to receive STI testing during the follow-up period. Perceptions that testing providers would think they were having risk behaviors because of PrEP use was significantly and negatively associated with the dependent variable (AOR: 0.51, 95% CI: 0.31, 0.86, *p* = 0.01). (Table 3 and Table 4).

## 4. Discussion

Consistent with previous studies targeting MSM PrEP users [15,16,17,18,19], our participants reported a high and stable prevalence of sexual risk behaviors (e.g., CAI, multiple male sex partnerships and SDU) over the study period. According to the CDC’s clinical practice guideline [21], they should receive STI testing at least every three months. However, there was a significant gap between the real situations and the recommendation, as only 62.7% and 47.8% of participants reported STI testing uptake at baseline and Month 3. Such prevalence was similar to that reported by MSM PrEP users in the United States [16,24]. Over one third of the participants who had taken up STI testing were screened to be positive, and majority of the STI cases were incident infection. Given the low STI testing uptake, it is possible some asymptomatic STI cases were not identified and linked to treatment and care. STI may threaten the sexual health of “PrEP tourists” and their sex partners. Moreover, the risk of STI transmission among this group of PrEP users was also high. Therefore, it is important to promote regular STI testing among MSM “PrEP tourists” and to open up STI testing for this population especially. In addition, PrEP users should receive renal function testing at least every 3 months based on the CDC guideline [21]. However, only 75.5 and 52.2% followed such recommendations at baseline and during the follow-up period. It is also important to increase regular renal function screening among “PrEP tourists” to ensure their safety.

NGO providing HIV prevention services in Hong Kong stand in a good position to provide and promote STI testing services for MSM “PrEP tourists”. On one hand, these NGO have good access to local MSM and are the main providers of HIV and STI testing services for this group [35]. On the other hand, some of these NGO are actively working with local and overseas private clinics to promote PrEP use among MSM [36]. It is possible to establish a service model involving NGO, PrEP clinics and public or private STI clinics to facilitate STI testing and treatment for PrEP users. In the long-term, a one-stop service for PrEP users should be considered, which provides rapid, walk-in PrEP care, and STI testing and treatment. The Dean Street Express Clinic in London, United Kingdom is a successful example [37].

The findings provided empirical insights for developing future rigorous and theory-based interventions. Participants who received STI testing at baseline were more likely to take up STI testing again at Month 3. It is likely that these participants with experience of STI testing were more familiar with STI testing services and likely to develop a habit of regular testing. Therefore, interventions promoting STI testing should start earlier at PrEP initiation. At baseline, less than half of the participants perceived a high or very high chance of taking up STI testing in the next three months. Similar to previous findings, behavioral intention to take up STI testing was a strong predictor of actual behavior [38]. More attention should be given to less motivated MSM “PrEP tourists” in future STI testing promotion programs. Motivational interviewing (MI) is a potential useful tool in future health promotion. It is a client-centered, non-directive, goal-oriented counseling technique, which helps the clients to explore and resolve any ambivalence that might have to change, especially among less motivated clients [39]. The United States CDC lists MI among its best evidence interventions [40]. A systematic review indicated that MI was acceptable to MSM and feasible to deliver over the phone [41]. With training, staff of NGO in Hong Kong had successfully implemented MI in promoting HIV testing among MSM [30]. Despite the high prevalence of sexual risk behaviors, only 12.7% of participants perceived a high or very high chance of STI. It is clear that the majority of them underestimated their risk of STIs. Perceived susceptibility to STI was associated with higher STI testing uptake during the follow-up period. Health communication messages about high risk of STI among MSM “PrEP tourists” across countries should be disseminated to the participants. A personalized STI risk self-assessment tool may be helpful to increase perceived susceptibility, which can be adapted from the “HIV risk calculator” developed by Chen & Dowdy [42]. About 30% of participants concerned about being stigmatized by a testing service due to PrEP use. Such concern was associated with lower STI testing uptake during the follow-up period. Previous studies showed that PrEP-related stigma was an important barrier hindering people at risk of HIV to use PrEP-related services [12]. Therefore, health promotion should also cover testing service providers to increase their understanding of PrEP. PrEP should be seen as a health option and a socially acceptable strategy for HIV prevention. Developing innovative STI testing models are also useful to address concerns related to stigma. Recently, a novel HIV self-testing service with online real-time counseling via live-chat applications has been implemented by local NGO. Such a service was effective in increasing HIV testing coverage and ensuring linkage to care [28,30]. It is possible to adapt the service model to STI testing.

This was one of the first studies investigating STI testing among PrEP tourists. However, it had a number of limitations. First, we were only able to involve two out of five clinics in Bangkok providing PrEP to MSM from Hong Kong. The costs of obtaining PrEP and services are similar across these clinics. Although MSM PrEP tourists may obtain PrEP from different PrEP clinics in Thailand, it is likely that they are facing some common difficulties when they are using the medication in Hong Kong. We believe information obtained from this project is important for developing new supportive services. Second, the sample size of this study was small and was not supported by sample size planning. Only 67 participants completed both the baseline and follow up surveys. This was a pilot study providing some preliminary data among this vulnerable group of PrEP users. Third, self-reported data might be subject to social desirability bias, as participants were likely to over-report HIV and other STI testing uptake, and to under-report their sexual risk behaviors. The real situations might be even worse. Fourth, we did not document which type of STI testing the participants carried out. Fifth, items measuring perceptions related to STI testing were self-constructed and were not validated by other studies. Moreover, we did not include the HBM construct of perceived severity due to the limited length of the questionnaire. Furthermore, we were not able to obtain the characteristics of PrEP tourists who refused to join the study. Thus, selection bias might exist. Finally, attrition bias might exist. Those who were bisexual and reported SDU at baseline were more likely to drop out of the study. However, sexual orientation and SDU were not associated with STI testing uptake in this study.

## 5. Conclusions

Despite the high risk of STI, MSM “PrEP tourists” reported low STI testing uptake. It is important to promote regular STI testing in this group and open sexual health care services to PrEP tourists, for STI testing and PrEP care.

## Figures and Tables

**Table 1:** Baseline characteristics of the participants.

	All Participants (n = 110)	Being Followed up at Month 3 (n = 67)	Loss to Follow-Up (n = 43)	*p* Value
	n (%)	n (%)	n (%)	
**Socio-demographics**				
Age group				
18–30	36 (32.7)	20 (29.9)	16 (37.2)	
31–40	55 (50.0)	36 (53.7)	19 (44.2)	
>40	19 (17.3)	11 (16.4)	8 (18.6)	0.61
Hong Kong permanent resident				
No	39 (35.5)	24 (35.8)	15 (34.9)	
Yes	71 (64.5)	43 (64.2)	28 (65.1)	0.92
Ethnicity				
Chinese	75 (68.2)	44 (65.7)	31 (72.1)	
White	30 (27.3)	19 (28.4)	11 (25.6)	
Southeast Asian	5 (4.5)	4 (6.0)	1 (2.3)	0.61
Relationship status				
Currently single	60 (54.5)	32 (47.8)	28 (65.1)	
Married/cohabiting with a man	50 (45.5)	35 (52.2)	15 (34.9)	0.07
Education level				
Senior high or below	9 (8.2)	7 (10.4)	2 (4.7)	
College or above	101 (91.8)	60 (89.6)	41 (95.3)	0.28
Monthly personal income				
≤HK $30,000 (US $3846.2)	38 (34.5)	24 (35.8)	14 (32.6)	
HK $30,001–50,000 (US $3846.3–6410.3)	32 (29.1)	24 (35.8)	8 (18.6)	
>HK $50,000 (US $6410.3)	40 (36.4)	19 (28.4)	21 (48.8)	0.06
Sexual orientation				
Homosexual	100 (90.9)	66 (98.5)	34 (79.1)	
Bisexual	8 (7.3)	1 (1.5)	7 (16.3)	
Refused to disclose	2 (1.8)	0 (0.0)	2 (4.7)	0.002
Duration of PrEP use (months)				
>12	36 (32.7)	24 (35.8)	12 (27.9)	
7–12	19 (17.3)	9 (13.4)	10 (23.3)	
1–6	12 (10.9)	8 (11.9)	4 (9.3)	
0	43 (39.1)	26 (38.8)	17 (39.5)	0.55
HBV vaccination				
No	21 (19.1)	12 (17.9)	9 (20.9)	
Yes	89 (80.9)	55 (82.1)	34 (79.1)	0.69
**HIV, HBV and/or HCV, other STI and renal function testing 2 weeks prior to baseline survey**				
HIV testing				
No	0 (0.0)	0 (0.0)	0 (0.0)	
Yes	110 (100.0)	67 (100.0)	43 (100.0)	1.00
HBV and/or HCV testing				
No	63 (57.3)	40 (59.7)	23 (53.5)	
Yes	47 (42.7)	27 (40.3)	20 (46.5)	0.52
Other STI testing				
No	41 (37.3)	25 (37.3)	16 (37.2)	
Yes	69 (62.7)	42 (62.7)	27 (62.8)	0.99
Renal function testing				
No	27 (24.5)	16 (23.9)	11 (25.6)	
Yes	83 (75.5)	51 (76.1)	32 (74.4)	0.84
**Sexual behaviors in the last 3 months**				
Number of RP with anal intercourse				
0	16 (14.5)	9 (13.4)	7 (16.3)	
1	56 (50.9)	36 (64.3)	20 (46.5)	
2–5	32 (29.1)	19 (28.4)	13 (30.2)	
>5	6 (5.5)	3 (4.5)	3 (7.0)	0.86
Number of NRP with anal intercourse				
0	18 (16.4)	10 (14.9)	8 (18.6)	
1	14 (12.7)	11 (16.4)	3 (7.0)	
2–5	43 (39.1)	26 (38.8)	17 (39.5)	
>5	35 (31.8)	20 (29.9)	15 (34.9)	0.52
CAI with men				
No	15 (13.6)	12 (17.9)	3 (7.0)	
Yes	95 (86.4)	55 (82.1)	40 (93.0)	0.10
SDU				
No	65 (59.1)	45 (67.2)	20 (46.5)	
Yes	45 (40.9)	22 (32.8)	23 (53.5)	0.03
**Perceptions related to STI testing**				
Perceived risk of contracting STI (% high/very high)	14 (12.7)	8 (11.9)	6 (14.0)	0.76
Mean (SD)	2.8 (0.8)	2.7 (0.8)	2.9 (0.6)	0.14
Taking up STI testing regularly (i.e., every 3 months) when using PrEP could detect STI infection earlier, so as to have better treatment outcomes (% agree/strongly agree)	104 (94.5)	63 (94.0)	41 (95.3)	0.77
Mean (SD)	4.3 (0.6)	4.2 (0.5)	4.4 (0.7)	0.14
Providers of STI testing services would think you are having risk behaviors because you are using PrEP (% agree/strongly agree)	35 (31.8)	21 (31.3)	14 (32.6)	0.89
Mean (SD)	2.8 (1.1)	2.8 (1.1)	2.8 (1.2)	0.84
Taking up STI testing regularly when using PrEP is troublesome for you (% agree/strongly agree)	32 (29.1)	23 (34.3)	9 (20.9)	0.13
Mean (SD)	2.6 (1.1)	2.8 (1.1)	2.4 (1.1)	0.051
People who are important to you suggest you taking up STI testing regularly when using PrEP (% agree/strongly agree)	84 (76.4)	50 (74.6)	34 (79.1)	0.59
Mean (SD)	4.0 (0.7)	4.0 (0.7)	4.0 (0.7)	0.96
It is easy for you to take up STI testing regularly when using PrEP of you want to (% agree/strongly agree)	91 (82.7)	54 (80.6)	37 (86.0)	0.46
Mean (SD)	3.9 (0.7)	3.9 (0.7)	4.0 (0.7)	0.40
Chance of taking up STI testing in the next 3 months (% high/very high)	59 (53.6)	32 (47.8)	27 (62.8)	0.12
Mean (SD)	3.3 (1.4)	3.2 (1.3)	3.6 (1.4)	0.20

**Table 2:** HIV, HBV and/or HCV, other STI and renal function testing, methods of PrEP use and sexual behaviors during the follow-up period measured at Month 3 (among participants being followed up at Month 3, n = 67).

	N	%
HIV testing		
No	0	0.0
Yes	67	100.0
HBV and/or HCV testing		
No	63	94.0
Yes	4	6.0
Other STI testing		
No	35	52.2
Yes	32	47.8
Renal function testing		
No	32	47.8
Yes	35	52.2
Methods of using PrEP		
Daily PrEP	47	70.1
On-demand PrEP	18	26.9
Episodic PrEP	2	3.0
Number of RP with anal intercourse		
0	7	10.4
1	39	58.2
2–5	18	26.9
>5	3	4.5
Number of NRP with anal intercourse		
0	9	13.4
1	13	19.4
2–5	37	55.2
>5	8	11.9
CAI with men		
No	13	19.4
Yes	54	80.6
SDU		
No	38	56.7
Yes	29	43.3

**Table 3:** Associations of background characteristics and STI testing uptake during the follow-up period (among participants being followed up at Month 3, n = 67).

	OR (95% CI)	*p* Values
**Socio-demographics**		
Age group		
18–30	1.0	
31–40	1.88 (0.62, 5.69)	0.27
>40	0.86 (0.19, 3.92)	0.84
Hong Kong permanent resident		
No	1.0	
Yes	0.51 (0.19, 1.42)	0.20
Ethnicity		
Chinese	1.0	
White	2.73 (0.90, 9.28)	0.08
Southeast Asian	4.77 (0.46, 49.62)	0.19
Relationship status		
Currently single	1.0	
Married/cohabiting with a man	1.07 (0.41, 2.80)	0.89
Education level		
Senior high or below	1.0	
College or above	0.66 (0.14, 3.19)	0.60
Monthly personal income		
≤HK $30,000 (US $3846.2)	1.0	
HK $30,001–50,000 (US $3846.3–6410.3)	1.18 (0.38, 3.67)	0.77
>HK $50,000 (US $6410.3)	1.06 (0.32, 3.56)	0.92
Sexual orientation		
Homosexual	1.0	
Bisexual	N.A.	N.A.
Duration of PrEP use (months)		
>12	1.0	
7–12	3.5 (0.60, 20.4)	0.16
1–6	0.33 (0.06, 2.00)	0.23
0	0.73 (0.24, 2.24)	0.59
HBV vaccination		
No	1.0	
Yes	0.90 (0.26, 3.13)	0.86
**HIV, HBV and/or HCV, other STI and renal function testing 2 weeks prior to baseline survey**		
HIV testing		
No	1.0	
Yes	N.A.	N.A.
HBV and/or HCV testing		
No	1.0	
Yes	1.32 (0.50, 3.50)	0.58
Other STI testing		
No	1.0	
Yes	2.83 (1.00, 8.01)	0.049
Renal function testing		
No	1.0	
Yes	1.24 (0.40, 3.83)	0.71
**HIV, HBV and/or HCV, and renal function testing during the follow-up period**		
HIV testing		
No	1.0	
Yes	N.A.	N.A.
HBV and/or HCV testing		
No	1.0	
Yes	N.A.	N.A.
Renal function testing		
No	1.0	
Yes	2.22 (0.83, 5.92)	0.11
**Sexual behaviors in the last 3 months measured at baseline**		
Number of RP with anal intercourse		
0	1.0	
1	0.45 (0.10, 2.07)	0.30
2–5	0.45 (0.09, 2.35)	0.34
>5	N.A.	N.A.
Number of NRP with anal intercourse		
0	1.0	
1	1.94 (0.32, 11.76)	0.47
2–5	2.72 (0.57, 12.91)	0.21
>5	2.33 (0.47, 11.69)	0.30
CAI with men		
No	1.0	
Yes	2.07 (0.55, 7.70)	0.28
SDU		
No	1.0	
Yes	0.50 (0.18, 1.43)	0.20
**Sexual behaviors in the last 3 months measured at Month 3**		
Number of RP with anal intercourse		
0	1.0	
1	0.34 (0.06, 1.99)	0.23
2–5	0.20 (0.03, 1.35)	0.10
>5	N.A.	N.A.
Number of NRP with anal intercourse		
0	1.0	
1	1.71 (0.29, 9.99)	0.55
2–5	2.11 (0.46, 9.74)	0.34
>5	2.00 (0.28, 14.20)	0.49
CAI with men		
No	1.0	
Yes	1.08 (0.32, 3.65)	0.90
SDU		
No	1.0	
Yes	0.50 (0.19, 1.33)	0.16
**Methods of PrEP use during the follow-up period**		
Daily PrEP	1.0	
On-demand PrEP	1.04 (0.35, 3.08)	0.94
Episodic PrEP	N.A.	N.A.

OR: crude odds ratios. CI: confidence interval, N.A.: not applicable.

**Table 4:** Associations between perceptions related to STI testing and STI testing uptake during the follow-up period (among participants being followed up at Month 3, n = 67).

	OR (95% CI)	*p* Values	AOR (95% CI)	*p* Values
Perceived risk of contracting STI	1.98 (1.03, 3.83)	0.04	1.90 (1.00, 3.75)	0.047
Taking up STI testing regularly (i.e., every 3 months) when using PrEP could detect STI earlier, so as to have better treatment outcomes	2.26 (0.88, 5.80)	0.09	1.94 (0.75, 5.04)	0.17
Providers of STI testing services would think you are having risk behaviors because you are using PrEP	0.50 (0.30, 0.82)	0.01	0.51 (0.31, 0.86)	0.01
Taking up STI testing regularly when using PrEP is troublesome for you	1.01 (0.66, 1.55)	0.96	1.04 (0.67, 1.61)	0.88
People who are important to you suggest you taking up STI testing regularly when using PrEP	1.90 (0.92, 3.95)	0.08	1.68 (0.79, 3.56)	0.18
It is easy for you to take up STI testing regularly when using PrEP of you want to	0.63 (0.32, 1.24)	0.18	0.59 (0.29, 1.20)	0.15
Chance of taking up STI testing in the next 3 months	1.75 (1.17, 2.62)	0.007	1.62 (1.05, 2.50)	0.03

OR: crude odds ratios. AOR: adjusted odds ratios, odds ratios adjusted for significant background characteristics listed in Table 3. CI: confidence interval.

## Data Availability

The data presented in this study are available on request from the corresponding author. The data are not publicly available as they contain sensitive personal behaviors.
